# Home sleep apnea test for patients undergoing atrial fibrillation ablation: An alternative for polysomnography?

**DOI:** 10.1002/joa3.12899

**Published:** 2023-07-22

**Authors:** Akihiro Sato, Hiroki Matsumoto, Takatoshi Kasai

**Affiliations:** ^1^ Department of Cardiovascular Biology and Medicine Juntendo University Graduate School of Medicine Tokyo Japan; ^2^ Cardiovascular Respiratory Sleep Medicine Juntendo University Graduate School of Medicine Tokyo Japan; ^3^ Sleep and Sleep‐Disordered Breathing Center Juntendo University Hospital Tokyo Japan

Editorial comment to “Sleep apnea severity in patients undergoing atrial fibrillation ablation: Home sleep apnea‐test and polysomnography comparison.”.^1^

Sleep apnea (SA) is associated with an increased incidence of cardiovascular diseases such as hypertension, coronary heart disease, heart failure and atrial fibrillation (AF). This indicates a possible cause‐and‐effect relationship between them. In general, SA is classified as obstructive or central based on respiratory effort. In patients with obstructive SA (OSA), repetitive upper airway obstruction during sleep leads to intermittent nocturnal hypoxemia, hypercapnia, overactivation of the sympathetic nervous system, enhanced oxidative stress and inflammation. These factors contribute to atrial remodeling and fibrosis, predisposing patients to an increased risk of AF. Catheter ablation (CA) is an effective treatment for AF; however, its relatively high recurrence rate remains an important issue. Although the recurrence rate of AF after CA is significantly higher in patients with OSA than in those without, optimal CPAP therapy is associated with a reduction in AF recurrence rates after CA. A meta‐analysis of nine prospective cohort studies including 2,134 participants indicated that CPAP is associated with a 37% relative risk reduction in AF recurrence among OSA patients under rhythm‐control strategies.^2^ Therefore, diagnosing and treating SA are important for patients with AF undergoing CA.

The Centers for Medicare & Medicaid Services (CMS) in the United States defines the indications of CPAP for OSA as an apnea‐hypopnea index (AHI) ≥15 or an AHI ≥5 with documented symptoms of excessive daytime sleepiness, impaired cognition, mood disorders or insomnia, or documented cardiovascular diseases.^3^ Notably, for the indication of CPAP in CMS, the AHI values measured using the home sleep apnea test (HSAT) can be considered equivalent to those measured using polysomnography (PSG), although the HSAT mentioned here is not the same as the devices used in Japan for HSAT. On the other hand, the current Japanese health insurance system defines CPAP indications as an AHI ≥20 assessed using PSG or an AHI ≥40 assessed using HSAT.^4^ Therefore, AF patients with an AHI <40 as indicated by HSAT must undergo additional PSG to determine CPAP indications. PSG is the standard diagnostic tool for SA; however, many patients suspected to have SA may not be able to undergo this test because of its high cost, limited access and long waiting times. Additionally, patients may be unwilling to undergo PSG without objective data suggesting pathological OSA. Patients with AF and OSA are less likely to complain of excessive daytime sleepiness, which is an important symptom indicating the need for treatment. Therefore, objective data suggestive of pathological OSA are important, particularly in patients with AF. The advantages of HSAT over PSG in terms of cost‐effectiveness have already been reported. Kawakami et al. reported the cost‐effectiveness of facilitating OSA screening using HSAT before CA in AF patients.^5^ In their study, the incremental cost‐effectiveness ratio of the Japanese health insurance system was compared among patients without screening, those with HSAT‐guided screening and those with PSG‐guided screening. HSAT‐guided screening was most cost‐effective at a willingness‐to‐pay threshold of JPY 5,000,000. However, few studies have investigated the validity of OSA severity derived using HSAT against that derived using PSG among patients with AF.

In this issue of the Journal, Tanaka et al.^1^ provided valuable insights into the correlation between the AHI derived using HSAT and that obtained using PSG in patients with AF. Prior to CA, they performed HSAT using a peripheral arterial tonometry (PAT)‐based device (Watch‐PAT200U [WP]; Itamar Medical Ltd.). WP devices were sent to the patients' homes, where the patients self‐administered the devices and returned them with a filled screening questionnaire. Afterward, they were admitted for CA. PSG was performed under stable postoperative conditions at least 1 day after CA. A significant correlation was observed between WP‐AHI and PSG‐AHI (*r* = 0.48, *p* < 0.001), and the best cut‐off value of the WP‐AHI predicting PSG‐AHI ≥20 (indications for CPAP as per the Japanese health insurance system), was almost identical to PSH‐AHI (i.e., 18.1). According to the results, patients deemed eligible for CPAP treatment by PSG (i.e., PSG‐AHI ≥20) were 70.9%, whereas patients deemed eligible for CPAP treatment by HSAT (i.e., WP‐AHI ≥40) were only 12.5%. This indicates that many patients who need to be treated with CPAP miss the indication if they do not undergo PSG.

Several limitations, some of which have been acknowledged by the authors themselves, should be considered when interpreting the study results. First, WP and PSG were not performed simultaneously; WP was performed before CA, and PSG was performed after CA. Therefore, CA may have influenced PSG‐AHI and caused some differences between WP‐AHI and PSG‐AHI. Second, the PAT‐based device indirectly detects apnea and hypopnea by selectively measuring peripheral arterial volume changes using a finger‐mounted plethysmogram. In patients with AF, irregular heartbeats may influence beat‐to‐beat changes in the peripheral arterial volume. Therefore, it is unclear whether these findings apply to other HSAT devices.

Nevertheless, the findings of the study by Tanaka et al. provide valuable information for cardiologists which will help them consider whether to initiate CPAP or perform PSG prior to or soon after CA in patients with a WP‐AHI ≥20. Ideally, the CPAP indication for patients with AF undergoing CA under the Japanese health insurance system is the same as that in CMS (i.e., the AHI values measured using HSAT can be treated as if they were measured using PSG). If so, more patients with AF with moderate‐to‐severe OSA will be treated with CPAP, AF recurrence in such patients will decrease and burden of healthcare costs may be minimized.

## FUNDING INFORMATION

This study was supported by JSPS KAKENHI (grant numbers: JP17K09527, JP18K15904, JP21K08116 and JP21K16034), a grant to The Intractable Respiratory Diseases and Pulmonary Hypertension Research Group from the Ministry of Health, Labor and Welfare (20FC1027) and a research grant from the Japanese Center for Research on Women in Sport, Juntendo University. These funding sources did not play any other role in this study.

## CONFLICT OF INTEREST STATEMENT

Drs. Akihiro Sato and Takatoshi Kasai are affiliated with a department endowed by Philips Japan, ResMed and Fukuda Denshi. The authors have no conflicts of interest to declare.
